# Postharvest Diseases of Pomegranate: Alternative Control Means and a Spiderweb Effect

**DOI:** 10.3390/jof9080808

**Published:** 2023-07-30

**Authors:** Annamaria Mincuzzi, Ugo Picciotti, Simona Marianna Sanzani, Francesca Garganese, Lluís Palou, Rocco Addante, Marco Ragni, Antonio Ippolito

**Affiliations:** 1 Department of Soil, Plant, and Food Sciences, University of Bari Bari Aldo Moro, Via Amendola 165/A, 70126 Bari, Italy; ugo.picciotti@uniba.it (U.P.); simonamarianna.sanzani@uniba.it (S.M.S.); francesca.garganese@uniba.it (F.G.); rocco.addante@uniba.it (R.A.); marco.ragni@uniba.it (M.R.); antonio.ippolito@uniba.it (A.I.); 2 Department of Marine Science and Applied Biology, University of Alicante, 03690 Alicante, Spain; 3 Pathology Laboratory, Postharvest Technology Center (CTP), Valencian Institute of Agrarian Research (IVIA), CV-315, Km 10.7, Montcada, 46113 Valencia, Spain; palou_llu@gva.es

**Keywords:** *Punica granatum*, gray mold, black heart, spiderweb, fungicide, fungistatic, Arachnida, biostimulants, microorganisms, diseases

## Abstract

The pomegranate is a fruit known since ancient times for its beneficial properties. It has recently aroused great interest in the industry and among consumers, leading to a significant increase in demand. Consequently, its cultivation has been boosted all over the world. The pomegranate crop suffers considerable yield losses, especially at the postharvest stage, because it is a “minor crop” with few permitted control means. To control latent (*Alternaria* spp., *Botrytis* spp., *Coniella* spp., *Colletotrichum* spp., and *Cytospora* spp.) and wound (*Aspergillus* spp., *Penicillium* spp., and *Talaromyces* spp.) fungal pathogens, different alternative compounds, previously evaluated in vitro, were tested in the field on pomegranate cv. Wonderful. A chitosan solution, a plant protein hydrolysate, and a red seaweed extract were compared with a chemical control treatment, all as preharvest (field application) and postharvest treatments and their combinations. At the end of the storage period, the incidence of stamen infections and external and internal rots, and the severity of internal decay were evaluated. Obtained data revealed that pre- and postharvest application of all substances reduced the epiphytic population on stamens. Preharvest applications of seaweed extract and plant hydrolysate were the most effective treatments to reduce the severity of internal pomegranate decays. Furthermore, the influence of spider (*Cheiracanthium mildei*) cocoons on the fruit calyx as a possible barrier against postharvest fungal pathogens was assessed in a ‘Mollar de Elche’ pomegranate organic orchard. Compared to no-cocoon fruit (control), the incidence of infected stamens and internal molds in those with spiderwebs was reduced by about 30%, and the mean severity of internal rots was halved. Spiderwebs analyzed via Scanning Electron Microscopy (SEM) disclosed a layered, unordered structure that did not allow for the passage of fungal spores due to its mean mesh size (1 to 20 µm ca). The aims of this research were (i) to evaluate alternative compounds useful to control postharvest pomegranate decays and (ii) to evaluate the effectiveness of spiders in reducing postharvest fungal infections by analyzing related mechanisms of action. Alternative control means proposed in the present work and calyx spider colonization may be helpful to reduce postharvest pomegranate diseases, yield losses, and waste production in an integrated control strategy, satisfying organic agriculture and the planned goals of Zero Hunger Challenge launched by the United Nations.

## 1. Introduction

The demand for functional foods, such as tea, pomegranates, algae, etc., is strongly increasing due to health benefits derived from dietary intake [1]. These properties are mainly related to active secondary metabolites such as alkaloids, terpenoids, and polyphenols, constituting a complex mixture of compounds involved in various biological functions of plants [2]. Mainly, pomegranate (*Punica granatum* L.) fruit are rich in polyphenolic compounds (gallotannins, ellagitannins, and pelletierine) and well-known for their antioxidant, antimicrobial, and antidiarrheal properties [3,4]; hence, the world request for fresh and processed pomegranates in recent years has a continuously increasing trend [5]. Sicily and Apulia regions are the primary internal market suppliers of Italian pomegranates, the production of which was over 25,000 tons in 2022 [6]; however, not all the production reaches the consumption since spoilage of different origins is an important issue for this minor crop [7]. Proper preharvest management and optimal storage conditions could extend the postharvest life of this appreciated fruit [8,9,10]. About 65% of yield losses are caused by latent pathogenic fungi, such as *Alternaria*, *Botrytis*, *Coniella*, *Colletotrichum,* and *Cytospora,* infecting the host during blooming or fruit-set in the tree [7,11]. The leftover percentage is related to wound fungal pathogens infecting fruit through lesions caused by bad handling, insects, and abiotic damages; according to pathogen etiology, species within *Aspergillus*, *Penicillium,* and *Talaromyces* genera are included in this group [7,11]. In Italy, despite the growing economic importance of this crop, few fungicides or alternative compounds are authorized to control fungal diseases. Until last year, as needed, employment of conventional and alternative fungicides was locally and yearly allowed according to regional legislation [12]. Among chemical compounds, in 2023, field and postharvest application of boscalid and fludioxonil, respectively, has been approved; similarly, among alternative control means, essential oils (geraniol, thymol, eugenol) and beneficial microorganisms (*Coniothyrium minitans*, *Trichoderma* species) have been allowed [13]. Nevertheless, all these allowed things are not enough to fulfill organic agriculture needs and aims requested by the United Nations. Indeed, several alternative compounds have been tested worldwide, such as crab shell chitosan [14], hot water and aqueous solutions of various salts [15], and wastewater extracts from olive milling [16], but results were not always encouraging. This research aimed to evaluate (i) the alternative compounds useful to control postharvest pomegranate decays and (ii) the influence of spider cocoons within the calyx on the reduction in postharvest fungal infections, analyzing for the latter putative-related mechanisms of action.

## 2. Materials and Methods

### 2.1. Alternative Compound Trial

#### 2.1.1. Experimental Scheme

The field trial was conducted for two consecutive years (2017 and 2018) in an orchard of pomegranates cv. Wonderful, located in Taranto province (Apulia region, southern Italy). Several alternative control means were tested according to the manufacturer’s recommendations [7]. The applied compounds were as follows: (i) lupin protein hydrolysate (AgricostanD^©^, Costantino and C. spa, Favria, Torino, Italy); (ii) liquid chitosan (ChitoPlant Solution^®^, Agritalia, Villa Saviola, Mantova, Italy); (iii) liquid fermented extract of Rhodophyta, iodine-rich, and fertilizer made of phosphorus and potassium (Red Bloc SW amended with Elfo, ICAS srl, Milano, Italy). Water was used as a negative control, while a formulate based on fludioxonil (50% of the active ingredient; Scholar, Syngenta Crop Protection AG, Syngenta Group Co. Ltd., Basel Switzerland) was applied as standard chemical control.

Three different application protocols were compared to evaluate the best application strategy: (i) preharvest treatment; (ii) pre- and postharvest treatments; and (iii) postharvest dipping. Preharvest trials were arranged in a completely randomized block design with three replicates containing three plants each. Plants were selected for growth uniformity and absence of evident symptoms of diseases and disorders and were sprayed (5 L plant^−1^, 20 hL ha^−1^) using a commercial motor-driven back sprayer (Fox Motori mod. 320, Poviglio, Re, Italy). Treatments were performed for three weeks, starting from the optimal blooming stage until fruit-set, focusing on pomegranate flower calyxes or just-set fruit. Untreated pomegranates of the same orchard were utilized in postharvest dipping assay. Apparently-healthy mature fruit were harvested as per marketing standards [17] and treated within 24 h from harvest to reduce non-specific infections and avoid chain discontinuity. Concerning postharvest treatments, pomegranates were 2-min dipped in each alternative compound and then air-dried. For pre- and postharvest combined application, according to each thesis, fruit already treated in the field were dipped in an alternative compound solution. Ten fruit, single-layer arranged for each replicate, were placed in a cardboard box in a micro-perforated plastic bag (Decco Magic Bag, Decco Italia S.r.l., Catania, Italy). Five boxes were used as replicates for each treatment, block, and administration combination. Pomegranate boxes were stored in optimal cold storage conditions at 7 ± 1 °C and 90–95% relative humidity (RH) for four months.

#### 2.1.2. Epiphytic Population of Stamens

During cold storage, the epiphytic population of stamens was assessed bimonthly; for each treatment and replicate, two fruit were randomly selected, and twelve stamens per fruit were plated on a semi-selective Potato Dextrose Agar (PDA; Conda, Madrid, Spain) amended with 250 mg L^−1^ of both streptomycin and ampicillin (Sigma-Aldrich, St. Louis, MI, USA). Three technical replicates per fruit were incubated at 24 ± 1 °C for five days in the dark. According to Mincuzzi et al. [11] and Barnett and Hunter [18], colonies were morphologically identified at the genus level. The number of colonies (CN) was rated to the total amount of stamens (SN) per plate (P) to evaluate the epiphytic population (EP). Final values were expressed as percentages as follows:

EP = [(CN/SN)/P] ×100.

EP values were averaged among technical replicates, and, considering technical replicates and randomized blocks, standard deviations were appraised. Sampled fruit were marked and replaced in the box, maintaining the original position.

#### 2.1.3. Evaluation of Rots

At the end of the storage period, the incidence of internal and external decay was assessed. External rot incidence was evaluated by the presence of lesions on the fruit surface, with or without sporulation. Pomegranates were cut longitudinally to assess internal rot incidence, and internal mold development was evaluated by means of an empirical scale with seven classes: from 0 (healthy fruit) to 6 (more than 75% infected area). Results were expressed as percentages, except for average severity.

### 2.2. Spiderweb Effect Trial

During various surveys in organic pomegranate orchards of Apulia and Basilicata regions (Italy), spider cocoons belonging to the spider species *Cheiracanthium mildei* (L. Koch, 1864) were observed within the calyx of fruit; stamens inside those calyxes appeared without apparent signs of fungal colonization. Hence, we planned to evaluate the putative effectiveness of spiderweb in preventing postharvest pomegranate rots ascribable to field infections of fungi. The trial was conducted in an organic orchard of pomegranates cv. Mollar del Elche located in the Bernalda area (Matera, Basilicata region, Italy). Based on the above-mentioned marketing standard, mature fruit with spider cocoons within the calyx and no-cocoon fruit were harvested and grouped into two different batches. Five replicates, each one made of 20 fruit, were collected per every batch, packaged, and cold-stored, as explained above.

### 2.3. Data Analysis

The statistical software package Prism9 for Mac (GraphPad Software Inc., Dotmatics, San Diego, CA, USA) was used to assess the incidence of infected stamens and external symptoms and the severity of internal rots. Percentage data of incidence and severity were statistically analyzed by ANOVA. In the case of homogeneity of variance, data from repeated experiments were combined. Significant differences (*p* ≤ 0.05) were identified by Tukey’s Multiple Comparisons test (TMC).

### 2.4. Scanning Electron Microscopy of Spiderwebs

To clarify the mechanisms involved in the putative cocoon effect, webs collected in October from pomegranate calyxes sampled in the above-mentioned ‘Mollar de Elche’ orchard were examined for their structure by Scanning Electron Microscopy (SEM; HITACHI TM 3000 Tabletop, Tokyo, Japan). SEM made it possible to observe the meshes and spatial distribution of the spiderweb spun by *C. mildei*. The spider webs were fixed onto a specimen stub (ø 12.5 mm, stub length 8 mm; Agar Scientific Ltd., Stansted, UK) with proper carbon double-sided tape [19]. After fixing, spiderwebs were metalized by an ion sputter coater (Edwards S150 Ion Sputter Coater, Crawley, UK) with gold/palladium for approximately 2 min at a pressure of 10 mA to improve the observation of presumably dielectric samples. The spiderweb observations were in a relative 3 µPa vacuum and 15 kV acceleration voltage.

## 3. Results

### 3.1. Alternative Compound Trial

**Figure 1:**
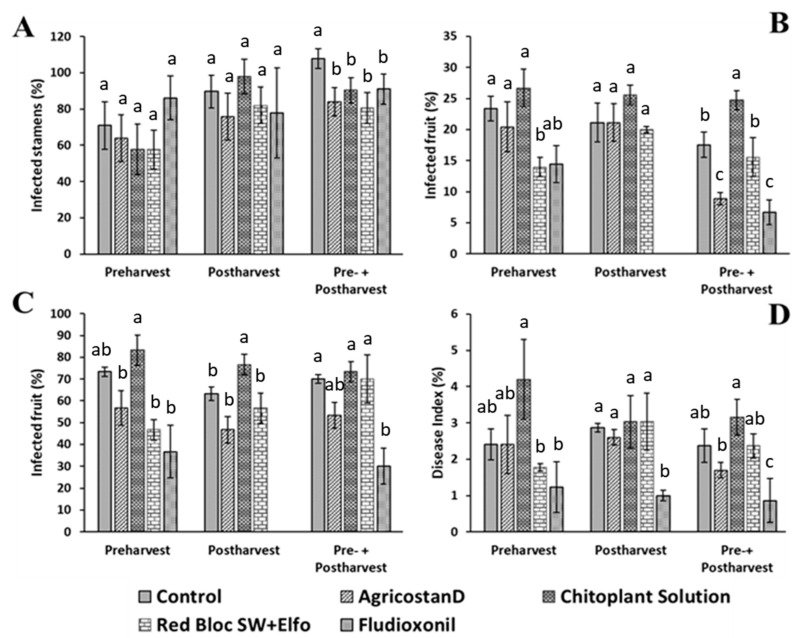
Alternative compound trial. Pomegranate fruit cv. Wonderful were sampled after 4 months of storage at 7 ± 1 °C and 90–95% RH. (**A**) Percentage of infected stamens. (**B**) Percentage of external rots. (**C**) Percentage of internal rots. (**D**) Severity of internal rots expressed according to disease index empirical scale. Bars represent the standard errors of triplicated datasets (*n* = 15). For each parameter, columns with different letters are statistically different according to TMC (*p* ≤ 0.05).

At the end of storage, the double application (both in pre- and postharvest) of all alternative compounds was effective in reducing the epiphytic stamen population as compared to the control (Figure 1A); reduction in infected stamens ranged between 14 and 25% with a performance not different than that of chemical control, whereas the single pre- or postharvest application was not effective. Stamens were mainly colonized by species belonging to *Penicillium* and *Talaromyces* genera, and other colonies were ascribed to *Alternaria* spp., *Botrytis* spp., *Coniella* spp., and *Cladosporium* spp.

Considering the external rot incidence (Figure 1B), Red Bloc SW + Elfo appeared to be the most effective treatment, showing a 39% reduction in preharvest treatments, comparable to that of chemical control. In postharvest application, none of the alternative compounds resulted effective in controlling rot incidence. The combined pre- and postharvest application gave the best results since the AgricostanD^©^ application led to a 50% reduction in rots, comparable to fludioxonil applied only before harvest.

Internal rot evaluation showed a trend comparable to that of the external rots (Figure 1C). When applied at preharvest, AgricostanD^©^ and Red Bloc SW + Elfo displayed a positive trend in reducing rot incidence by 22 and 36%, respectively, although not significantly different. If double-applied, AgricostanD^©^ displayed a positive trend with an average reduction of 30%, although the decrease was not statistically relevant. In double treatments, a better performance was demonstrated by fludioxonil (57% reduction) as compared to the control.

The severity assessment of internal rots overlapped with the trend of the analyses mentioned above (Figure 1D). Among all treatments, fludioxonil resulted in the most effective treatment; instead, with alternative compounds, no significant differences were observed.

### 3.2. Spiderweb Effect Trial

**Figure 2:**
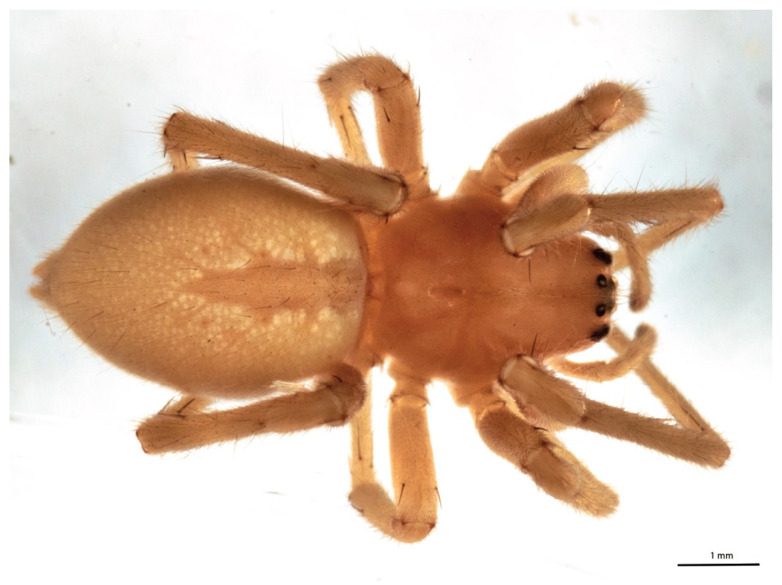
An immature female of *Cheiracanthium mildei* located in the cocoons of the calyx of pomegranate fruit in Bernalda (Basilicata region, southern Italy).

**Figure 3:**
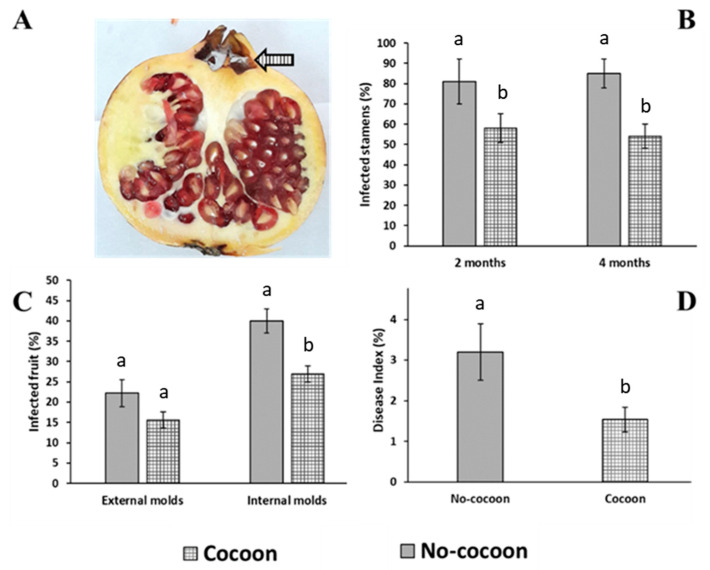
Spiderweb effect trial. Pomegranate fruit cv. Mollar de Elche stored at 7 ± 1 °C and 90–95% RH showing (**A**) a *Cheiracanthium mildei* nest within the pomegranate calyx (arrow). (**B**) The percentage of infected stamens was evaluated after 2 and 4 months of cold storage; instead. (**C**) The percentage of external and internal rots was assessed after 4 months of cold storage. (**D**) Severity of internal rots, expressed according to the disease index empirical scale, was evaluated at the end of storage. Bars represent the standard errors of triplicated datasets (*n* = 15). For each parameter, columns with different letters are statistically different according to TMC (*p* ≤ 0.05).

After 2 and 4 months of cold storage, the incidence of infected stamens on pomegranates exhibiting the spiderweb cocoon of *C. mildei* (Figure 2) within the calyx (Figure 3A) was reduced by 28 and 37%, respectively, as compared to fruit with no cocoons (Figure 3B). Fungi isolated from stamens mainly belonged to *Penicillium* and *Talaromyces* genera. Even though external decays were not statistically reduced, the internal rot incidence was reduced by 34% on fruit having spider cocoons (Figure 3C). Finally, the severity on fruit with cocoons was about half compared to the control (Figure 3D).

### 3.3. Scanning Electron Microscopy of Spiderwebs

**Figure 4:**
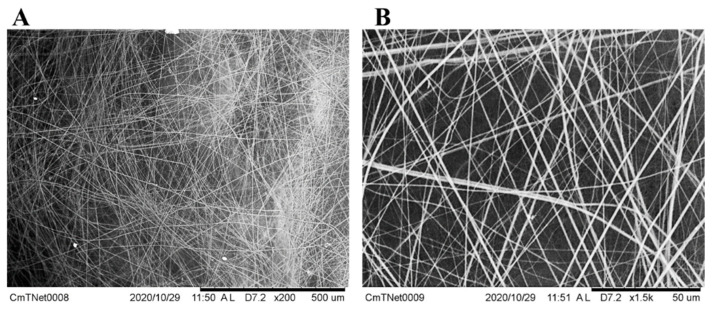
Scanning Electron Microscopy (SEM) images of *Cheiracanthium mildei* web. The web has (**A**) a layered, unordered structure, (**B**) the silk threads that compose it are ca. 2 µm in diameter, and meshes of the lattice structure range between 1 to 20 µm, on average.

SEM analysis showed that the *C. mildei* spiderweb had a layered, unordered structure (Figure 4). The silk threads that compose the spider web were ca. 2 µm in diameter (Figure 4B). The layering of the web formed a lattice structure with meshes varying from 1 to 20 µm ca. (Figure 4B).

## 4. Discussion

Maintaining optimal storage conditions and health could extend the shelf life and, thus, pomegranate availability [9,10] since there are gaps in world pomegranate production between growing seasons. Indeed, pomegranates are chiefly available from September to February in the northern hemisphere and from April to July in the southern one; therefore, pomegranates are globally scarce in March and August [20]. Morphological features of pomegranate fruit, such as the persistence of the calyx, remains, and senescent stamens, allow for the setting of latent infections [11,12,21], as reported for strawberries and table grapes [22,23]. Indeed, this is one of the leading causes of pomegranate losses, as described by Opara et al. [24,25], who analyzed the South African pomegranate trade. The same research highlighted the importance of avoiding abiotic stresses, pests, and fungal infections in the field and fruit damage during harvesting, postharvest handling, and transport to reduce the incidence of secondary fungal infections [8,26]. Most of the fungal infections of pomegranates occur in the field during the blooming and early fruit-growing stages, remaining latent until optimal conditions for pathogen growth [11,12]. As latent infections are difficult to control, specific strategies must be implemented for prevention [7,11]. However, since pomegranate is considered a minor crop, few treatments are allowed by the current legislation, also in Italy. Thomidis [27] tested several molecules to control pathogenic fungi on ‘Wonderful’ Greek pomegranates; except for thiophanate methyl and tebuconazole, preharvest sprays with cyproconazole, azoxystrobin, and a mix of pyrachlostrobin-boscalid were not effective in controlling postharvest decays [27]. Failure to control postharvest disease was probably ascribable to the late application time (1 or 2 weeks before harvest); indeed, fruit already harbored latent infections that occurred months earlier during the blooming stage.

On the other hand, postharvest application could be effective in reducing primary infections caused by wound pathogens and secondary infections related to nesting propagation and moldy stamens. The importance of application timing according to the target pathogens was highlighted by Pala and colleagues [28], who evaluated, for two consecutive years, the same fungicides in two different scheduled programs; particularly, spraying pomegranates mainly during the blooming stage was more effective in controlling *Alternaria alternata* and *Coniella granati* [28]. These latent pathogens infect pomegranates chiefly during anthesis [11] after overwintering in mummies [29]. These facts are also confirmed by our data: incidence and severity of postharvest rots, generally, are significantly reduced by repeated treatments in the field. Being registered as a postharvest fungicide and effective in reducing crown rots caused by latent and wound pathogens [12,30,31], in our trials, fludioxonil was selected as positive chemical control, and its efficacy was fully confirmed. D’Aquino et al. [30] analyzed chemical residues of fludioxonil postharvest application, highlighting their absence in the edible portion of the fruit; furthermore, in the USA, the Environmental Protection Agency (EPA) gave a complete registration tolerating residues of 5 ppm [32]. Kishore [33] evaluated various fungicides and validated the effectiveness of captan and copper oxychloride, but the latter scarred the pomegranate rind due to copper phytotoxicity, reducing market acceptability. Until 2021, copper was allowed to be used in conventional and organic farming in Italy if maximum dosages and application times were respected [34], although bioaccumulation risk and toxicity for plants, animals, and humans are well-known.

Chitosan is a D-glucosamine linear polymer, mainly obtained by alkaline deacetylation of chitin extracted from different sources [12], indeed classified as a basic substance [35]. Such factors as pH, deacetylation degree (60 to 100%), molecular weight (3800 to 20,000 Da), origin, and active ingredient concentration affect its solubility in water, bio-adhesive properties, and above all, antifungal properties [36,37]. Chitosan has been often used as a coating to preserve arils during cold storage [38,39,40], but also it significantly reduced the in vitro growth of fungi attacking pomegranate [7] and the incidence [31] and severity [41] of pomegranate postharvest decays. The synergistic application of chitosan and essential oils (lemongrass, thyme, and oregano) that exhibited antimicrobial properties [41] was tested to improve chitosan’s effectiveness. Nevertheless, the in vivo use of chitosan on pomegranates appeared inadequate, as confirmed by our data obtained in trials with ‘Wonderful’ fruit. The epiphytic fungal population of pomegranate stamens treated with chitosan was reduced; however, chitosan-treated fruit showed the highest incidence and severity of external and internal rots. ChitoPlant Solution^®^ effectiveness was limited to the stamens and external tissues, probably due to its direct activity against the main pomegranate postharvest pathogens, as proved by previous in vitro assays disclosing a radial growth reduction of 70% [7]. Having the formulation of a 12% proportion of chitosan, the induction of resistance, and the film-forming properties, which are well-known modes of action of chitosan [37], were probably not sufficient to limit infection development due to the final low concentration of the active ingredient used at the recommended label dosage.

Furthermore, physical and enzymatic processes during fruit ripening, over the months elapsing before harvest, might cause chitosan molecule degradation, worsening its effectiveness [42]. Probably, under these conditions, chitosan may act as a resistance inducer [43]. It might be interesting to test repeated treatments and/or higher chitosan concentrations aiming at an efficacy enhancement.

Concerning protein hydrolysates, generally, AgricostanD^©^ significantly reduced the incidence and severity of pomegranate rots, mainly if applied at preharvest or at pre- plus postharvest. Being obtained by hydrolysis of protein-rich organic matter, protein hydrolysates are abundant in peptides and amino acids (e.g., glutamic and aspartic acids, alanine, methionine, and proline) that enhance plant protection mechanisms. Moreover, they are carriers of scarcely available nutritive elements such as iron, boron, calcium, and magnesium, also well-known biostimulants [44]. Detailing, AgricostanD^©^ is an OGM-free protein hydrolysate of lupines enriched in oligosaccharides. This compound improves plant nutrient uptake by promoting growth, reducing fertilization needs, and increasing the resistance to biotic and abiotic stresses. Substances belonging to the same group of compounds, such as soy, lupin, and casein hydrolysates, were successfully employed to control the growth of *B. cinerea* both in vitro and in vivo [44]; notably, disease incidence of artificially infected table grapes was reduced by 67 and 54% by soy and casein hydrolysates, respectively. On naturally infected grapes, soy hydrolysate was the best treatment in the field and after harvest [44]. According to our trials, if applied both at pre- and postharvest, AgricostanD^©^ reduced epiphytic stamen populations by 22% and external rot incidence by 50%. The double application of lupin protein hydrolysate better stimulated fruit defenses, considering the lower severity of internal fruit rots.

Biostimulants made from seaweeds, especially Rhodophyta (red algae) and Ochrophyta-Phaeophyceae (brown algae), were widely used to stimulate antioxidant production, improve stress and disease tolerance, and regulate gene expression providing systemic resistance in plants [45]. Red Bloc SW is a liquid extract obtained from the fermentation of red algae enriched in iodine, which induces the production of phytoalexins and has healing properties; according to the manufacturer’s leaflet, qualitative features (as total soluble solids and color) of fruit can be improved by its administration. In our trials, Red Bloc SW was applied in combination with Elfo, a fertilizer made of phosphoric anhydride and potassium oxide, substances capable of stimulating plant defenses and improving resistance to disease caused by fungal pathogens [46]. Our investigation confirmed the effectiveness of this algal fertilizer; if applied both at preharvest and pre- and postharvest, the extract, in combination with Elfo, reduced the incidence of disease caused by pathogenic fungi colonizing the stamens, showing a preventative and curative effect. Application of Red Bloc SW + Elfo in the field significantly reduced both external and internal rot incidence, probably related to the induction of resistance, which is an effective method to control latent infections; also, disease severity was significantly lower compared to the control. Recent investigations showed some direct inhibitory effects of Red Bloc SW on *B. cinerea*, *Monilinia* spp., and *Penicillium expansum* (Ligorio A., unpublished results).

Nevertheless, repeated antifungal treatments are needed because of the staggered and prolonged pomegranate bloom and the morphological features of the fruit, which make it challenging to reach the inner part of the flowers/fruitlets by traditional spraying systems. This scenario stresses the importance of new reliable control means. Hence, in the field, employing biocontrol agents could simplify the control of postharvest decays. Particularly, during the field sampling and the cold storage monitoring, spider’s nests were observed within the pomegranate calyx: the number of cocoons and/or nursery nests in pomegranates observed in the field was lower than the number observed during the cold storage surveys; nests built by *C. mildei* were used for overwintering. This spider, widespread and characteristic of the Mediterranean area, is common in vineyards [47]. *C. mildei* could cross gaps between plants through own-constructed silk bridges, and it hides, nests, and lays eggs in bunches of grapes, preferring plants with reduced bark and fields subjected to low chemical treatments, in which the preys are abundant; it principally eats leafhoppers and caterpillars, but probably also thrips and mites [48]. All these described features are ascribable to organic pomegranate orchards, too, where the trees are low-cork and detached enough from each other; insects and mites are abundant, especially during flowering and fruiting stages, and, above all, the pomegranate calyx is a suitable nest hollow-like grape bunch. In addition, being capable of surviving at low temperatures (having enough environmental humidity), it can survive during pomegranate cold storage. The presence of adults and juveniles of *C. mildei* occurred from blooming until postharvest of pomegranate fruit [49]. The observed cocoon is a white dense cotton-like bundle interwoven to stamens. Examined specimens caught from cold-stored fruit were mainly males, even if discovered fertile females (Addante R., personal communication). Calyx-held cocoon was clearly visible, allowing the selection of ‘Mollar de Elche’ pomegranates to arrange the trial. Our data displayed a significantly reduced incidence of infected stamens and internal rots for the cocoon batch; similarly, fruit with spider’s nests showed a lower disease severity than the control. Finding a comfortable and satisfying environment, *C. mildei* built nests and cocoons; the average mesh size suggests that the spiderwebs’ mesh does not permit the passage of spores of primary pathogenic fungi, whose conidia measure an average of 11.54 × 7.2 µm [11]. Starting from the flowering stage, *C. mildei* web could act as a physical barrier, a filter, against the fungal conidia that colonized the flower tissues [11]. Indeed, each spider’s nest protects the calyx from primary latent fungal infections during the blooming stage, avoiding the entry of pathogenic propagules and thus reducing the colonization of senescent stamens, which can act as a putative source of secondary inoculum. Spiderwebs are known for physically blocking aerosol droplets with silk threads [50,51]. Moreover, spiderwebs are sticky, which is the reason why they can catch and seize conidia at a simple touch; therefore, *C. mildei*’s webs could physically block the conidia of the pathogenic pomegranate fungi, preventing them from contact with susceptible organs of the host plant, thus avoiding the infectious process of pomegranates. To the best of our knowledge, this is the first report on the effectiveness of spiderwebs in reducing fruit postharvest fungal diseases; indeed, until now, spiders were only used as biocontrol agents against pests [52,53,54], and no one tested their effectiveness as a means to control decay caused by fungal pathogens. This hypothetical and promising mechanical mode of action should be examined in depth to assess a new biological control strategy for postharvest diseases.

## 5. Conclusions

All tested compounds were suitable for lowering the fungal population of pomegranate stamens if applied at pre- and postharvest, reducing an important source of inoculum/infections. Field application and pre- and postharvest applications of both red seaweed fertilizer and lupin protein hydrolysate were the best combinations to reduce external and internal rot incidence and severity. Nevertheless, chitosan efficacy was limited to reducing the stamen epiphytic population. Fludioxonil confirmed its effectiveness if applied before harvest corroborating its marketability. Finally, spider’s nests and cocoons seem to be a new and promising solution to reduce the incidence and severity of fruit rots in organic agriculture and respect the One Health approach for the safety of plants, the environment, animals, and humans. The integrated approach in the field and postharvest treatments with alternative compounds or biocontrol agents, good agronomical practices, and optimal cold storage conditions remain the key points for early control of pomegranate postharvest diseases and losses.

## Data Availability

Not applicable.

## References

[1] Daliri E.B.M., Lee B.H., Liong M.T. (2015). Current Trends and Future Perspectives on Functional Foods and Nutraceuticals. Beneficial Microorganisms in Food and Nutraceuticals.

[2] Wink M. (2022). Current understanding of modes of action of multicomponent bioactive phytochemicals: Potential for nutraceuticals and antimicrobials. Annu. Rev. Food Sci. Technol..

[3] Brighenti V., Iseppi R., Pinzi L., Mincuzzi A., Ippolito A., Messi P., Sanzani S.M., Rastelli G., Pellati F. (2021). Antifungal Activity and DNA Topoisomerase Inhibition of Hydrolysable Tannins from *Punica granatum* L.. Int. J. Mol. Sci..

[4] Yang X., Niu Z., Wang X., Lu X., Sun J., Carpena M., Prieto M.A., Simal-Gandara J., Xiao J., Liu C. (2022). The Nutritional and Bioactive Components, Potential Health Function and Comprehensive Utilization of Pomegranate: A Review. Food Rev. Int..

[5] Caruso A., Barbarossa A., Tassone A., Ceramella J., Carocci A., Catalano A., Basile G., Fazio A., Iacopetta D., Franchini C. (2020). Pomegranate: Nutraceutical with promising benefits on human health. Appl. Sci..

[6] ISTAT. https://www.istat.it/.

[7] Mincuzzi A., Sanzani S.M., Caputo M., D’Ambrosio P., Palou L., Ippolito A. Effectiveness of alternative means for controlling pomegranate postharvest pathogens. Proceedings of the VI International Symposium on Postharvest Pathology: Innovation and Advanced Technologies for Managing Postharvest Pathogens.

[8] Murthy D.S., Gajanana T.M., Sudha M., Dakshinamoorthy V. (2009). Marketing and Post-Harvest Losses in Fruits: Its Implications on Availability and Economy. Marketing.

[9] Arendse E. (2014). Determining Optimum Storage Conditions for Pomegranate Fruit (cv. Wonderful). Master’s Thesis.

[10] Arendse E., Fawole O.A., Opara U.L. (2015). Effects of postharvest handling and storage on physiological attributes and quality of pomegranate fruit (*Punica granatum* L.). Int. J. Postharvest Technol. Innov..

[11] Mincuzzi A., Sanzani S.M., Palou L., Ragni M., Ippolito A. (2022). Postharvest Rot of Pomegranate Fruit in Southern Italy: Characterization of the Main Pathogens. J. Fungi.

[12] Mincuzzi A., Ippolito A., Kahramanoglu I. (2023). Pomegranate: Postharvest Fungal Diseases and Control. New Advances in Postharvest Technology.

[13] BDF. https://www.bdfsrl.it/.

[14] Munhuweyi K., Lennox C.L., Meitz-Hopkins J.C., Caleb O.J., Sigge G.O., Opara U.L. In vitro effects of crab shell chitosan against mycelial growth of *Botrytis* sp., *Penicillium* sp. and *Pilidiella granati*. Proceedings of the III International Symposium on Postharvest Pathology: Using Science to Increase Food Availability.

[15] Palou L., Taberner V. Evaluation of hot water and GRAS salt solutions for the control of postharvest gray and green molds of pomegranate fruit. Proceedings of the VI International Symposium on Postharvest Pathology: Innovation and Advanced Technologies for Managing Postharvest Pathogens.

[16] Quaglia M., Moretti C., Cerri M., Linoci G., Cappelletti G., Urbani S., Taticchi A. (2016). Effect of extracts of wastewater from olive milling in postharvest treatments of pomegranate fruit decay caused by *Penicillium adametzioides*. Postharvest Biol. Technol..

[17] UNECE. https://unece.org/sites/default/files/2022-09/ECE_CTCS_WP.7_2022_06_E.pdf.

[18] Barnett H.L., Hunter B.B. (1998). Illustrated Genera of Imperfect Fungi.

[19] Savino I., Campanale C., Trotti P., Massarelli C., Corriero G., Uricchio V.F. (2022). Effects and impacts of different oxidative digestion treatments on virgin and aged Microplastic particles. Polymers.

[20] Kahramanoglu I. (2019). Trends in pomegranate sector: Production, postharvest handling and marketing. Int. J. Agric. For. Life Sci..

[21] Mincuzzi A., Ippolito A., Montemurro C., Sanzani S.M. (2020). Characterization of *Penicillium ss* and *Aspergillus* sect. *nigri* causing postharvest rots of pomegranate fruit in Southern Italy. Int. J. Food Microbiol..

[22] Picciotti U., Araujo Dalbon V., Ciancio A., Colagiero M., Cozzi G., De Bellis L., Finetti-Sialer M.M., Greco D., Ippolito A., Lahbib N. (2023). “Ectomosphere”: Insects and Microorganism Interactions. Microorganisms.

[23] Habib W., Khalil J., Mincuzzi A., Saab C., Gerges E., Tsouvalakis H.C., Ippolito A., Sanzani S.M. (2021). Fungal pathogens associated with harvested table grapes in Lebanon, and characterization of the mycotoxigenic genera. Phytopathol. Mediterr..

[24] Opara I.K., Fawole O.A., Opara U.L. (2021). Postharvest losses of pomegranate fruit at the packhouse and implications for sustainability indicators. Sustainability.

[25] Opara I.K., Fawole O.A., Kelly C., Opara U.L. (2021). Quantification of on-farm pomegranate fruit postharvest losses and waste, and implications on sustainability indicators: South African case study. Sustainability.

[26] Sudharshan G.M., Anand M.B., Sudulaimuttu D. (2013). Marketing and post-harvest losses in fruits: Its implications on availability and economy-A study on pomegranate in Karnataka. Int. J. Manag. Soc. Sci. Res..

[27] Thomidis T. (2014). Fruit rots of pomegranate (cv. Wonderful) in Greece. Australas. Plant Pathol..

[28] Pala H., Tatli A., Yilmaz C., Özgüven A.I. (2009). Important diseases of pomegranate fruit and control possibilities in Turkey. Acta Hortic..

[29] Kumar A., Chahal T.S., Singh M.H.S., Kaur H., Rawal R. (2017). Studies of Alternaria black spot disease of pomegranate caused by *Alternaria alternata* in Punjab. J. Appl. Nat. Sci..

[30] D’Aquino S., Schirra M., Palma A., Angioni A., Cabras P., Gentile A., Tribulato E. Effectiveness of fludioxonil in control storage decay on pomegranate fruit. Proceedings of the I International Symposium on Pomegranate and Minor Mediterranean Fruits 818.

[31] Munhuweyi K., Lennox C.L., Meitz-Hopkins J.C., Caleb O.J., Sigge G.O., Opara U.L. (2017). Investigating the effects of crab shell chitosan on fungal mycelial growth and postharvest quality attributes of pomegranate whole fruit and arils. Sci. Hortic..

[32] EPA. https://www.federalregister.gov/documents/2005/05/18/05-9778/fludioxonil-pesticide-tolerance.

[33] Kishore K., Bhardwaj S.S., Jayant K. (2008). Field evaluation of fungicides against fruit spot and rot diseases of pomegranate. Haryana J. Hortic. Sci..

[34] 834/2007/CE. https://eur-lex.europa.eu/legal-content/IT/TXT/PDF/?uri=CELEX:02007R0834-20130701&from=BG.

[35] Marchand P.A. (2023). Evolution of plant protection active substances in Europe: The disappearance of chemicals in favour of biocontrol agents. Environ. Sci. Pollut. Res..

[36] Shahrajabian M.H., Chaski C., Polyzos N., Tzortzakis N., Petropoulos S.A. (2021). Sustainable agriculture systems in vegetable production using chitin and chitosan as plant biostimulants. Biomolecules.

[37] Romanazzi G., Moumni M. (2022). Chitosan and other edible coatings to extend shelf life, manage postharvest decay, and reduce loss and waste of fresh fruits and vegetables. Curr. Opin. Biotechnol..

[38] Varasteh F., Arzani K., Barzegar M., Zamani Z. (2012). Changes in anthocyanins in arils of chitosan-coated pomegranate (*Punica granatum* L. cv. Rabbab-e-Neyriz) fruit during cold storage. Food Chem..

[39] Ghasemnezhad M., Zareh S., Rassa M., Sajedi R.H. (2013). Effect of chitosan coating on maintenance of aril quality, microbial population and PPO activity of pomegranate (*Punica granatum* L. cv. Tarom) at cold storage temperature. J. Sci. Food Agric..

[40] Özdemir K.S., Gökmen V. (2017). Extending the shelf-life of pomegranate arils with chitosan-ascorbic acid coating. LWT-Food Sci. Technol..

[41] Kawhena T.G., Opara U.L., Fawole O.A. (2021). A comparative study of antimicrobial and antioxidant activities of plant essential oils and extracts as candidate ingredients for edible coatings to control decay in ‘Wonderful’ pomegranate. Molecules.

[42] Pandit A., Indurkar A., Deshpande C., Jain R., Dandekar P. (2021). A systematic review of physical techniques for chitosan degradation. Carbohydr. Polym. Technol. Appl..

[43] Garganese F., Sanzani S.M., Di Rella D., Schena L., Ippolito A. (2019). Pre-and postharvest application of alternative means to control *Alternaria* Brown spot of citrus. Crop Prot..

[44] Lachhab N., Sanzani S.M., Bahouaoui M.A., Boselli M., Ippolito A. (2016). Effect of some protein hydrolysates against gray mould of table and wine grapes. Eur. J. Plant Pathol..

[45] Nanda S., Kumar G., Hussain S. (2022). Utilization of seaweed-based biostimulants in improving plant and soil health: Current updates and future prospective. Int. J. Environ. Sci. Technol..

[46] ICAS. https://www.icasinternational.it/.

[47] Van Keer K., Van Keer J., Koninck H.D., Vanuytven H. (2007). Another Mediterranean spider, *Cheiracanthium mildei* L. Koch, 1864 (Araneae: Miturgidae), new to Belgium. Nieuwsbrief van de Belgische arachnologische Vereniging.

[48] Carroll D. (2012). Spiders in San Joaquin Valley Vineyards. Pests, Biters and IPM Agents. https://www.aaie.net/wp-content/uploads/2013/01/Spiders-in-San-Joaquin-ValleyVineyards-2012.pdf.

[49] Rozwałka R., Rutkowski T., Bielak-Bielecki P. (2017). New data on introduced and rare synanthropic spider species (Arachnida: Araneae) in Poland (II). Ann. Univ. Mariae Curie-Sklodowska Sect. C–Biol..

[50] Smither S.J., Piercy T.J., Eastaugh L., Steward J.A., Lever M.S. (2011). An alternative method of measuring aerosol survival using spiders’ webs and its use for the filoviruses. J. Virol. Methods.

[51] Mainelis G. (2020). Bioaerosol sampling: Classical approaches, advances, and perspectives. Aerosol Sci. Technol..

[52] García L.F., Núñez E., Lacava M., Silva H., Martínez S., Pétillon J. (2021). Experimental assessment of trophic ecology in a generalist spider predator: Implications for biocontrol in Uruguayan crops. J. Appl. Entomol..

[53] Cuff J.P., Tercel M.P., Drake L.E., Vaughan I.P., Bell J.R., OrozcoterWengel P., Müller C.T., Symondson W.O. (2022). Density-independent prey choice, taxonomy, life history, and web characteristics determine the diet and biocontrol potential of spiders (Linyphiidae and Lycosidae) in cereal crops. Environ. DNA.

[54] Manthen S.V., Mahindrakar Y.Y., Hippargi R.V. (2023). Ecology of spiders in pomegranate orchard: Implications for integrated pest management (IPM). Methodology.

